# The changes of distance between nipples following correction of women pectus excavatum

**DOI:** 10.1038/s41598-022-24768-4

**Published:** 2023-01-09

**Authors:** Gyeol Yoo, Hui Hyung Jeon, Eun Young Rha, Jun Gul Ko, Sang Oon Baek, Jun Yong Lee, Jin Yong Jeong

**Affiliations:** 1 Department of Plastic and Reconstructive Surgery, Incheon St. Mary’s Hospital, College of Medicine, The Catholic University of Korea, Seoul, Republic of Korea; 2 Department of Plastic and Reconstructive Surgery, Eunpyeong St. Mary’s Hospital, College of Medicine, The Catholic University of Korea, Seoul, Republic of Korea; 3 Department of Thoracic and Cardiovascular Surgery, Incheon St. Mary’s Hospital, College of Medicine, The Catholic University of Korea, 56, Dongsu-Ro, Bupyeong-Gu, Incheon, Seoul, 21431 Republic of Korea

**Keywords:** Medical research, Outcomes research

## Abstract

The breasts in women pectus excavatum patients frequently appear to be slanting medially along the inclination of the distorted ribs. This study aims to evaluate changes in the distance between the nipples and to find out whether medially slanting breasts are corrected in women pectus excavatum patients following modified Nuss procedure. This case series analysis enrolled 22 young women patients with pectus excavatum between October 2011 and September 2020. We measured all the patients’ distances from the sternal midline to the right and left nipples, based on chest computerized tomography. We calculated the distances between nipples as being the sum of the right and left distances. The mean age of patients was 16.50 ± 4.73 years, and the follow-up periods were 35.59 ± 20.23 months. The postoperative Haller indices (2.89 ± 0.43) were significantly lower than the preoperative Haller indices (5.14 ± 1.96) (*p* = 0.000). The distances between the nipples before and after Nuss procedure were 145.17 ± 17.73 mm and 172.29 ± 19.11 mm, which is a significant increase following surgery. (*p* = 0.000). Our results demonstrated that skeletal correction with modified Nuss procedure in pectus excavatum increased the distance between nipples, indicating that medially slanting breasts had been corrected.

## Introduction

Pectus excavatum is the most common congenital chest wall deformity, presenting a depression of the anterior chest wall associated with the inward curvature of the costal cartilages attached to sternal depressed areas. The incidence rate is reported to be around 1/400 live births^1,2^. Because men are afflicted five times more frequently than women, little attention has been directed to their special symptoms and the indications for surgical management in women pectus excavatum patients as compared to men. Recently, however, women patient cosmetic concerns have increased interest in chest wall deformities^3^. Chest wall depression becomes more severe during the period of rapid skeletal growth in early adolescence, causing women breast distortion^4^. Women pectus excavatum breasts appears to slant towards each other along the inclination of the distorted ribs. The authors noted that medially slanting breasts are simultaneously corrected with chest depression by pectus excavatum repair without any surgery at the breast tissue. However, only a few studies have examined the changes in breast distance before and after women pectus excavatum repair.

We examined whether there was a change in the distance between both breasts by comparing the distances both nipples before and after modified Nuss procedure.

## Results

**Figure 1:**
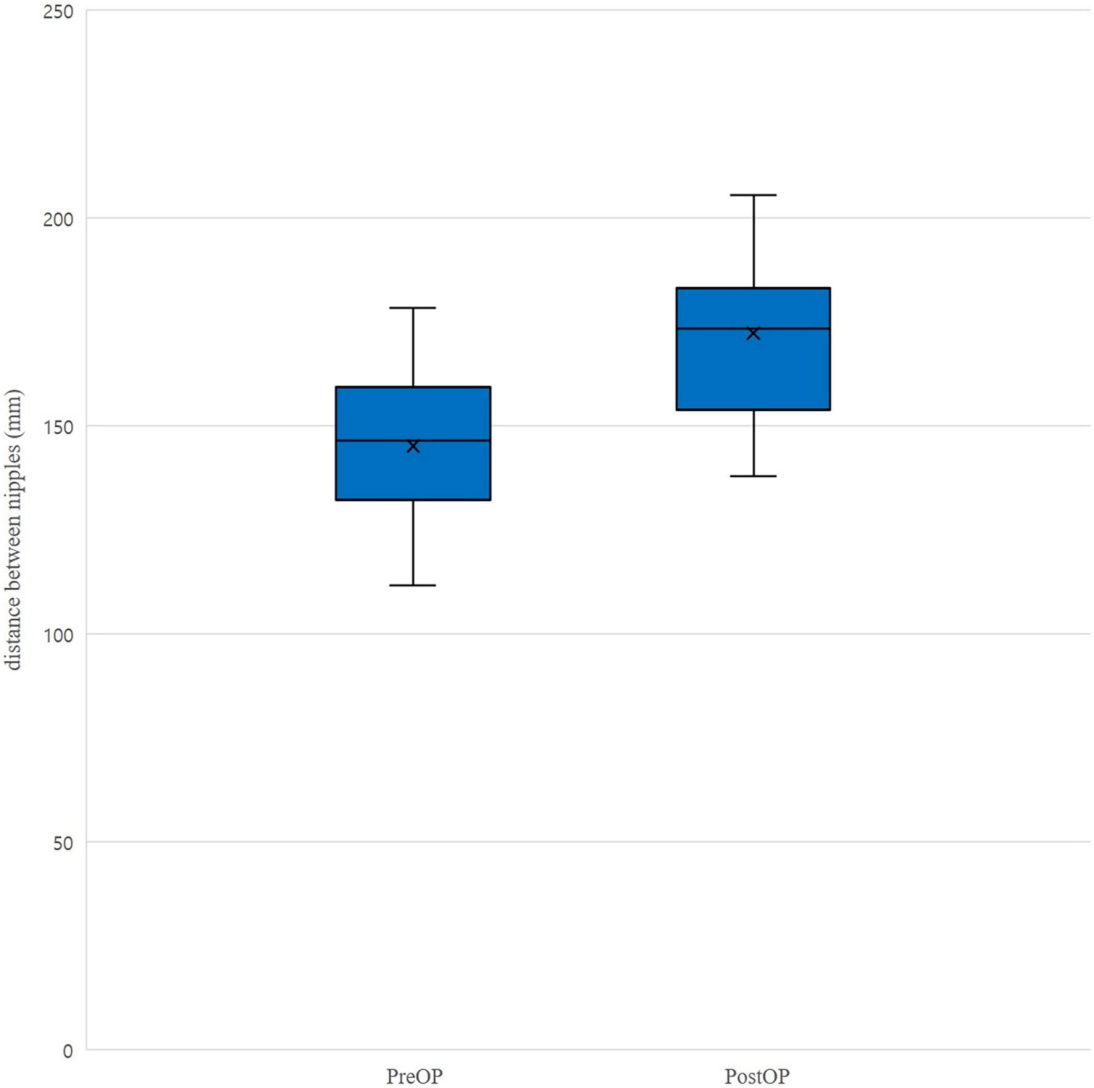
Box plot of the distance between nipples before and after modified Nuss procedures. Horizontal line indicates the median, box the interquartile range (IQR), whiskers extend to upper adjacent value (large value = 75th percentile + 1.5 × IQR) and lower adjacent value (smallest value = 25th percentile—1.5 × IQR), and ‘x’ in box represents the mean.

The mean age of patients was 16.50 ± 4.73 years, and the follow-up periods were at 35.59 ± 20.23 months. The preoperative Haller indices were 5.14 ± 1.96 (Table 1). All patients had needlescope-assisted three-point fixation of the pectus bar done, which is a modification of the Nuss procedure (n = 22). Quadrangular fixation of the pectus bars placed with needlescope-assisted three-point fixation was done in15 out of 22 patients. The postoperative Haller indices (2.89 ± 0.43) were significantly lower than the preoperative Haller indices (5.14 ± 1.96) (*p* = 0.000). The distance between the nipples was 145.17 ± 17.73 mm before modified Nuss procedure and 172.29 ± 19.11 mm following modified Nuss procedure, which is significant increase. (*p* = 0.000) (Table 2, Fig. 1).

**Table 1:** Patient characteristics.

	Average and SD
Age (years)	16.50 ± 4.73
Follow up (months)	35.59 ± 20.23
Haller index	5.14 ± 1.96

**Table 2:** Changes of the Haller index and distance between nipples before and after modified Nuss procedure.

	Before Nuss procedure	After Nuss procedure	*P* valule
Haller index	5.14 ± 1.96	2.89 ± 0.43	0.000
Distance between nipples (mm)	145.17 ± 17.73	172.29 ± 19.11	0.000

## Discussion

Pectus excavatum, which is the most common chest deformity, is characterized by a depression of the sternum and adjacent costal cartilages. Pectus excavatum may go unrepaired in childhood or adolescence because physicians often dismiss such patients as having an inconsequential problem instead of a cosmetic deformity^5^. Especially in women pectus excavatum, because of the special meaning of breasts for women as a symbol of femininity, cosmetic deformities are more problematic than in men. Many methods have been reported to correct them, such as augmentation with breast implants^6,7^, customized silastic implanting^8^, lipofilling^9^, cartilage chips grafting^10^, or local flaps^11^. These methods are used to provide cosmetic improvement. However, these procedures may not always achieve a sufficiently pleasing aesthetic result. Many women pectus excuvatum patients present with symptoms of cardiopulmonary dysfunction due to depressed anterior chest wall compressing the intrathoracic organs, such as the heart and lungs, in relation to the degree of deformity^12^. These symptoms also increased psychosocial stress, which can limit social activities^13^. Physiologic and cardiopulmonary symptoms have been the primary reason for pectus excavatum surgery in the majority of women pectus excuvatum patients, and cosmesis is also important to them^14^. It has been recommended to correct chest wall deformity in pectus excavatum patients and to correct aesthetic deformity. Various techniques, such as the Ravitch procedure^15^, Nuss procedure^16^, and their modifications^17^, had been used to correct pectus excavatum. In this study, the authors have performed needlescope-assisted three-point fixation^18^, with or without quadrangular fixation^19^ of the pectus bars which was a technique invented to avoid bar displacement, a common and serious complication of the Nuss procedure.

Schwabegger et al. named the symptom that normally developed breasts in puberty^20^ were slanting medially along the slope of distorted ribs in women pectus excavatum patients as breast strabismus^21–23^. These breast deformities like breast strabismus cause emotional distress in female pectus excuvatum patients, even though a functional issue with breast strabismus has not been known. By remodeling the anterior thoracic wall with Nuss procedure, the slanting breasts were relocated to their orthotopic position, resulting in a more naturally breast projection^23^. The author's experience also shows the same above-described pattern. All patients were satisfied with the changes of their breast shape after repair.

**Figure 2:**
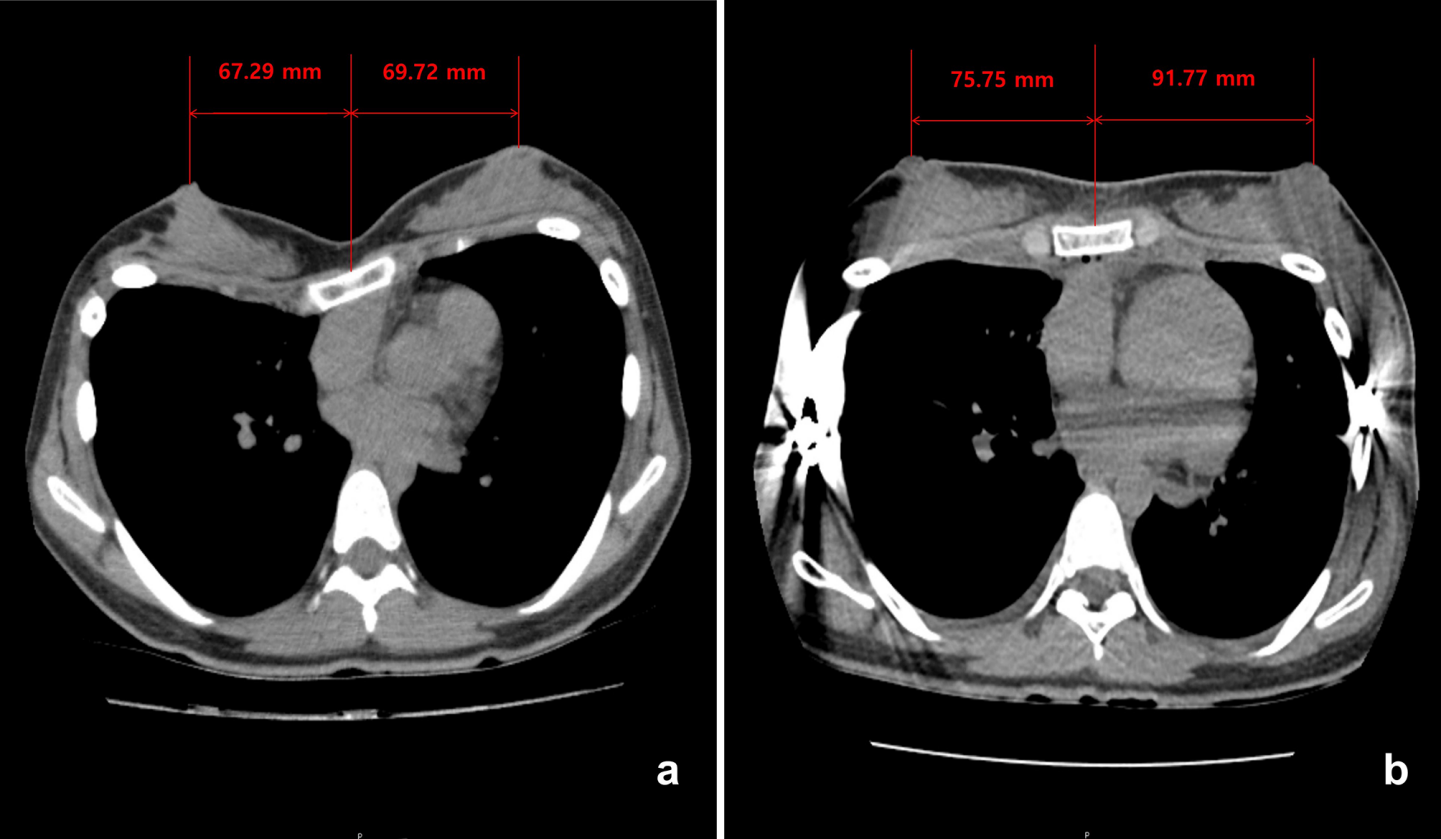
Nineteen year-old women pectus excavatum patient who had a modified Nuss repair. (**a**) The distance between nipples was 137.01 mm before modified Nuss procedure. (**b**) The distance between nipples was 167.52 mm after modified Nuss procedure.

Because the breast strabismus causes a diminished intermammary distance with strabismus of the nipple-areola complexes, reposition of the breasts to an aesthetically acceptable position in women pectus excavatum patients following Nuss procedure is seemed to show increasing the intermammary distance. However, there have been no studies of properly quantified intermammary distance changes before and after pectus excavatum repair in women pectus excavatum patients. Because the breast is a three-dimensional structure, that shape can be expressed with a variety of indicators. Although simple numerical values are insufficient as a means of the breast shape expression, the authors quantified the pre- and postoperative changes in the distance between nipples by measuring the distance between them on the chest CT sections (Fig. 2).

This study had several limitations. First, the study compared the distance between the nipples of patients with pectus excavatum using only CT images. Second, various parameters that affect intermammary distance were not assessed. Further research through indicators and means that can express three-dimensional changes more accurately will be required. Third, quantifying emotional distress for breast shapes including breast strabismus was not assessed. Further research for quantifying emotional distress before and after repair in women pectus excavatum will be needed.

Our results demonstrate that the skeletal correction with pectus excavatum repair with modified Nuss procedure results in an increased distance between nipples, demonstrating breast strabismus correction. This also suggests that skeletal correction alone, without breast surgery, might be useful for aesthetic improvement of medially slanting breast deformity in women pectus excavatum patients.

## Methods

### Study patients

We analysed the medical records and computed tomography (CT) findings of 22 selected patients who had been diagnosed with pectus excavatum at Incheon St. Mary’s Hospital between October 2011 and September 2020. The patients had needlescope-assisted three-point fixation^18^ done both with and without pectus bar quadrangular fixation^19^. This is modification of Nuss procedure. Inclusion criteria for this study were as follows: (1) adolescent and adult women, (2) having pectus excavatum with a Haller index > 3.25, (3) who had undergone chest CT before and after a modified Nuss procedure.

### Distance between the nipples

**Figure 3:**
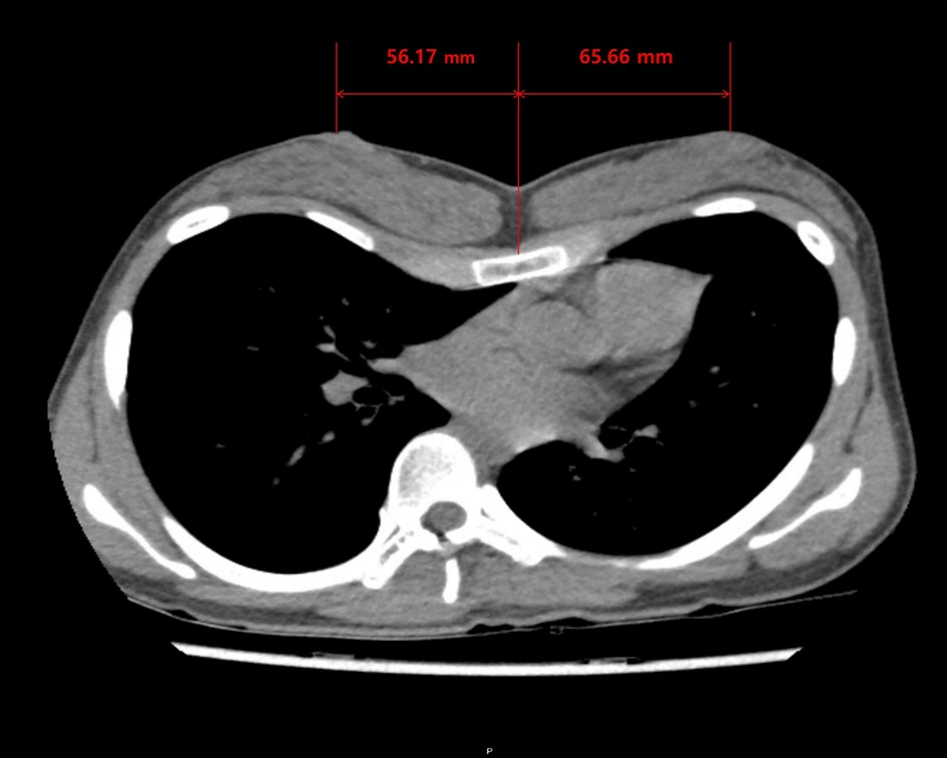
Measuring the distance between the nipples. Each distance from the sternal midline to the right and left nipples based on chest computerized tomography (CT) was measured and the distance between the nipples was calculated as the sum of the right and left distances.

Based on chest CT, we used the Picture Archiving Communication System (PetaVision, Seoul, Korea) to measure the distance between the sternal midline and the patients’ nipples on the left and right sides. We calculated the distance between the nipples by adding the right and left distances together (Fig. 3).

### Statistical analysis

Statistical analysis was performed using SPSS ver. 18 for Windows (SPSS, Chicago, IL, USA). All data is expressed as an average and standard deviation (SD). The paired t-test was used to compare differences between preoperative and postoperative parameters. A *P*-value of < 0.05 was considered to be statistically significant.

### Ethics approval

This study was approved by the Institutional Review Board of Incheon St. Mary’s Hospital, College of Medicine, the Catholic University of Korea (IRB approval number: OC21RASI0101). The study was performed in accordance with the Declaration of Helsinki. The requirement for informed consent was waived by the Institutional Review Board due to the retrospective study design.

## Data Availability

The datasets generated and analysed during the current study are available from the corresponding author on reasonable request.
